# Study on the Changes of Antioxidant System and Respiratory Metabolism in Rice Grains Under Nitrogen-Modified Atmosphere Storage from the Targeted Metabolomics Perspective

**DOI:** 10.3390/foods14213643

**Published:** 2025-10-25

**Authors:** Ming Chen, Xia Ma, Wenhao Li, Feiyan Xue, Chenling Qu

**Affiliations:** School of Food and Strategic Reserves, Henan University of Technology, Zhengzhou 450001, China; cm990315@163.com (M.C.); maxia2022@163.com (X.M.); liwenhao0730@163.com (W.L.); 17536357849@163.com (F.X.)

**Keywords:** nitrogen-modified atmosphere storage (N_2_-MAS), fatty acid values (FAV), reactive oxygen species (ROS), antioxidant enzymes, targeted-metabolomics

## Abstract

Nitrogen-modified atmosphere technology, due to its effectiveness in pest control, is widely used in grain storage as an eco-friendly preservation method. This study compared the quality changes in unhulled rough rice (paddy) stored under nitrogen-modified atmosphere and conventional conditions. Fatty acid value (FAV), reactive oxygen species (ROS) content, coenzyme levels, antioxidant enzyme activities, and concentrations of central carbon metabolism-related metabolites of paddy were monitored during storage under different storage conditions. The results revealed that compared to conventional storage, nitrogen-modified atmosphere resulted in lower FAV and ROS levels, as well as higher pyridine nucleotides contents and antioxidant enzyme activities, including superoxide dismutase (SOD), peroxidase (POD), catalase (CAT), and glutathione reductase (GR). Metabolomic profiling demonstrated that N_2_-MAS induced metabolic changes characterized by the down-regulation of 2-hydroxyglutaric acid and the up-regulation of fructose 6-phosphate, glucose 1-phosphate, glycerol 3-phosphate, gluconic acid, fumaric acid, and malic acid, which collectively contribute to reduced oxidative damage and enhanced preservation quality. These findings elucidated the mechanism of N_2_-MAS-delayed quality deterioration and revealed the regulatory role of the antioxidant system and central carbon metabolism.

## 1. Introduction

Rice (*Oryza sativa* L.) serves as a globally vital grain, providing essential nutrients such as proteins, carbohydrates, fats, and micronutrients to billions of people worldwide [1,2]. Agricultural production of grain exhibits cyclical harvest patterns influenced by seasonal and climatic variability, while grain is consumed on a daily basis [2]. Therefore, safe grain storage is a prerequisite for global food security [3]. However, during long-term storage, grain loss caused by the grain itself, pests, and microbial metabolic activities is inevitable, and these are regulated by abiotic factors (temperature, humidity and oxygen concentration) in the storage environment [4].

Currently, the most widely used rice storage methods primarily include conventional storage, low-temperature storage, and modified atmosphere storage [1,2]. Conventional storage requires that rice grains meet specific pre-storage criteria, including impurity content below 1% and moisture content lower than the safe level, sometimes followed by the application of techniques such as ventilation for temperature control and fumigation for insect control to ensure storage safety. Low-temperature storage refers to a grain storage technology that utilizes refrigeration equipment or natural low temperature air to maintain the storage environment at a cool level. It was shown that low-temperature storage conditions effectively maintained the abundance of cellular glycerophospholipids in rice grains, slowed the accumulation of free fatty acids, and thereby enhanced their intrinsic resistance to senescence during storage. However, this technology imposed stringent requirements on the thermal insulation and moisture resistance performance of grain storage warehouses [1].

Modified atmosphere storage technology has been widely implemented in postharvest preservation and pest control of agricultural products such as cereals, fruits, and vegetables to prolong shelf life [5]. The ambient gaseous composition during storage constitutes one of the most critical abiotic factors governing the metabolic activity of cereal grains [6]. It was reported that modified atmosphere storage with 98% nitrogen concentration effectively preserved antioxidant enzyme activity in wheat seeds and retarded the accumulation of oxidative damage [6].

Grain metabolism during storage was recognized as the primary contributor to nutrient depletion and oxidative damage [6,7]. Notably, studies demonstrated that the rise in reactive oxygen species (ROS), induced by lipid peroxidation and electron leakage in the mitochondrial respiratory chain, is a critical factor leading to the quality deterioration during grain storage [8]. The accumulation of ROS-induced damage is regulated by the pyridine nucleotide coenzyme system, wherein NADPH-dependent antioxidant system mitigates ROS-mediated oxidative stress while NADH maintains energy metabolism homeostasis [7].

As an important field within systems biology, metabolomics identifies small-molecule metabolites from metabolic pathways and discovers biomarkers relevant to physiological states [9]. Building on this technical foundation, Wang et al. utilized widely targeted metabolomics to elucidate geographical variations in metabolic profiles of daylily [10]. The ongoing metabolic activities during postharvest storage had been shown to fundamentally govern quality evolution patterns, establishing metabolomics as an indispensable tool for deciphering preservation mechanisms in grain storage [11]. Metabolomics analysis comparing MAS and CS rice grains revealed that MAS technology delayed quality deterioration by coordinately regulating amino acid metabolism, fatty acid metabolism, and carbohydrate metabolic pathways [2].

Therefore, to evaluate the effect of nitrogen-modified atmosphere storage (N_2_-MAS) on rice grains quality and elucidate its underlying mechanisms, this study investigated changes in fatty acid values (FAV), reactive oxygen species (ROS) contents, and NAD(H)/NADP(H) levels during rice grains storage. Targeted metabolomics was used to systematically analyze central carbon metabolism differences in rice grains stored under different conditions.

## 2. Materials and Methods

### 2.1. Rice Grain Materials and Storage Conditions

An indica rice ‘Lianhua Xiangsi’ was harvested from Jiangsu Province, China, which underwent cleaning and drying before use, with impurity content <1% and initial moisture content of 12.3%.

Two storage conditions were used in our experiments, including conventional storage (CS) and nitrogen-modified atmosphere storage (N_2_-MAS). CS: The cleaned rough rice grains samples were packaged into transparent open-top bags (2 kg per bag) and stored statically in a constant temperature and humidity incubator under controlled conditions of 25 °C ± 1 °C and 65% relative humidity (consistent with the modified atmosphere storage conditions). N_2_-MAS: Rough rice grains samples were stored in transparent sealed polyethylene bags filled with nitrogen. The residual oxygen level in the bags was monitored weekly with a gas detector (MIC-600, Shenzhen Yiyuntian Technology Co., Ltd., Shenzhen, China) and was maintained below 1.5 ± 0.1% throughout storage. These bags were all put in a constant temperature and humidity chamber maintained at 25 °C and 65 ± 5% relative humidity. The storage period lasted 180 days, with quality assessments performed every 90 days.

Prior to analysis, paddy samples were dehusked using a rice huller (THU35C-C, Satake Engineering Co., Ltd., Suzhou, China). Subsequently, the brown rice was promptly processed under low-temperature (4 °C) and light-protected conditions using a low-temperature continuous hammer cyclone mill (TDW-5000, Beijing Tongxin Tianbo Technology Development Co., Ltd., Beijing, China) with standardized grinding parameters (rotation speed: 12,000 rpm, duration: 1 min). The obtained brown rice flour was used for subsequent analysis.

### 2.2. Determination of Fatty Acid Values (FAV)

FAV was measured using the method by Qu et al. [2]. Initially, 10 g of brown rice flour was combined with 50 mL of anhydrous ethanol and subjected to shaking at 100 r·min^−1^ for 10 min. Subsequently, the mixture was filtered, and a 25 mL aliquot of the filtrate was diluted with 50 mL of carbon dioxide-free water (distilled water was heated to boiling and maintained for at least 10 min to remove dissolved carbon dioxide, then cooled to room temperature in a sealed container and used immediately). Following this, the diluted filtrate was titrated with a standardized potassium hydroxide (KOH) solution using phenolphthalein as an indicator. Three replicates were conducted for each sample. FAV was defined as the milligrams of KOH required to neutralize free fatty acids in 100 g of rice grains sample.

### 2.3. Determination of Reactive Oxygen Species (ROS) Contents

Brown rice flour was prepared according to the standardized procedure and immediately subjected to reactive oxygen species content analysis. The superoxide anion (O_2_^−^) content was determined using the superoxide anion activity content assay kit (BC1290, Beijing Solarbio Science & Technology Co., Ltd., Beijing, China). The O_2_^−^ content (µmol·g^−1^) was measured by absorbance at 530 nm. The H_2_O_2_ content was determined using the hydrogen peroxide (H_2_O_2_) content assay kit (BC3590, Beijing Solarbio Science & Technology Co., Ltd., Beijing, China). The H_2_O_2_ content (µmol·g^−1^) was measured by absorbance at 415 nm. Each sample was analyzed in triplicate.

### 2.4. Determination of Antioxidant Enzyme Activities

Preparation of enzyme extract: 0.1 g of brown rice powder was accurately weighed, mixed with a pre-cooled extraction buffer, and homogenized in an ice bath. The resulting homogenate was centrifuged at high speed at 4 °C. The supernatant collected served as the crude enzyme solution.

Superoxide dismutase (SOD) activity was determined using the SOD activity assay kit (BC0170, Beijing Solarbio Science and Technology Co., Ltd., Beijing, China), which was based on the inhibition of nitroblue tetrazolium (NBT) reduction. The xanthine and xanthine oxidase reaction system generated superoxide anions (O_2_^−^), which reduced NBT to produce blue formazan. The blue formazan exhibited maximum absorption at 560 nm. Superoxide dismutase (SOD) scavenges O_2_^−^, thereby inhibiting the formation of blue formazan. The SOD activity (U·g^−1^) was measured by absorbance at 560 nm. One unit of SOD enzyme activity (U·g^−1^) was defined as the amount required to achieved 50% inhibition per gram of sample in the xanthine oxidase-coupled reaction system. Each sample was analyzed in triplicate.

Peroxidase (POD) activity was assayed using the POD activity assay kit (BC0090, Beijing Solarbio Science and Technology Co., Ltd., Beijing, China), following the guaiacol oxidation method. The POD activity was determined based on the increase in absorbance at 470 nm. One unit of enzyme activity (U·g^−1^) was defined as the amount that caused a change of 0.01 in absorbance at 470 nm per minute per gram of sample. Each sample was analyzed in triplicate.

Catalase (CAT) activity was measured using the method of Qu et al. [2]. In this assay, an excess of H_2_O_2_ was introduced into the reaction system, and the residual substrate was titrated with KMnO_4_. Enzyme activity was determined based on the KMnO_4_ consumption required to neutralize unreacted H_2_O_2_, with triplicate measurements performed for each sample to ensure reproducibility. One unit of enzyme activity (U·g^−1^) was defined as the amount that catalyzed the degradation of 1 µmol H_2_O_2_ per minute per gram of sample in the reaction system.

Glutathione reductase (GR) activity was measured using the method by Olszewska-Czyz et al. [12]. In this assay, the oxidized glutathione (GSSG) was catalyzed to reduced glutathione (GSH), and NADPH served as the electron donor. The enzyme activity was determined based on the linear decrease in absorbance at 340 nm, which directly correlated with the oxidation rate of NADPH, with triplicate measurements performed for each sample to ensure reproducibility. One unit of enzyme activity (U·g^−1^) was defined as the amount that catalyzed the oxidation of 1 µmol NADPH per minute per gram of sample under conditions of 37 °C and pH 8.0.

### 2.5. Determination of Pyridine Nucleotides Contents

Quantification of pyridine nucleotides, including NAD^+^, NADH, NADP^+^, and NADPH, was performed following the protocol established by Lin et al. [13]. First, 0.2 g of brown rice flour was homogenized at 4 °C in 3 mL of 0.1 M HCl (for NAD^+^/NADP^+^ extraction) or 3 mL of 0.1 M NaOH (for NADH/NADPH extraction). The homogenate was subsequently heated in a boiling water bath for 5 min, followed by immediate cooling on ice, and centrifuged at 10,000× *g* for 10 min at 4 °C to collect the supernatant. Supernatants were neutralized with 0.1 M NaOH or HCl (adjusted to pH 7.0) and centrifuged again under the same conditions. Final supernatants were kept on ice for coenzyme assays.

Freshly prepared reaction mixtures (per sample) contained equal volumes of 1 M Tricine-NaOH buffer (pH 8.0), 40 mM ethylene diamine tetraacetic acid (EDTA), 4.2 mM 3(4,5-dimethylthiazol-2-yl)-2,5-diphenyltetrazolium bromide (MTT), 16.6 mM phenazine ethosulfate (PES), and 25 mM glucose-6-phosphate (for NADP^+^/NADPH) or 5 M ethanol (for NAD^+^/NADH). A volume of 300 µL of the mixture was combined with 50 µL of supernatant in a 1.5 mL microtube, and the solution was then brought to a final volume of 500 µL by adding 0.1 M NaCl. After pre-incubation at 37 °C for 3 min, 20 µL of G6PDH (for NADP^+^/NADPH) or ADH (for NAD^+^/NADH) was added to initiate enzymatic cycling. Reactions were terminated after 30 min by adding 300 µL of 6 M NaCl, followed by centrifugation (10,000× *g*, 10 min, 4 °C). The precipitate was dissolved in 2 mL of 95% ethanol, and absorbance was measured at 570 nm. NAD(P)(H) content was calculated as μmol·g^−1^ dry weight.

### 2.6. Targeted Metabolomics Profiling of Central Carbon Metabolism

#### 2.6.1. Metabolite Extraction and Standard Solution Preparation

The samples were ground at 60 Hz for 60 s into a dry powder. An aliquot of each individual sample was precisely weighed and transferred to an Eppendorf tube. Following the addition of two small steel balls and 500 µL of MeOH/H_2_O (3/1, *v*/*v*, pre-cooled to −40 °C), the samples were vortexed for 30 s. A three-cycle process of homogenization (4 min, 35 Hz) and ice–water bath sonication (5 min) was then performed. After that, the samples were incubated at −40 °C for 1 h and centrifuged (15 min, 12,000 rpm, 4 °C). From the resulting supernatant, 100 µL was taken and dried. The dried residue was redissolved in 250 µL of water. This reconstituted solution was vortexed, filtered through a membrane, and placed into vial inserts for HPLC-MS/MS analysis. QC sample was prepared by combining 100 µL from each sample.

#### 2.6.2. HPIC-MRM-MS Analysis

An HPLC system (Thermo Scientific Dionex ICS-6000, Waltham, MA, USA) was employed for separation using Dionex IonPac AS11-HC (2 × 250 mm) (Thermo Scientific, Waltham, MA, USA) and AG11-HC (2 × 50 mm) (Thermo Scientific, Waltham, MA, USA) columns. The mobile phases were 100 mM NaOH in water (A) and ultrapure water (D). An additional pumping system delivered a solution of 2 mM acetic acid in methanol, which was mixed with the column effluent at a flow rate of 0.15 mL·min^−1^ before ESI. The analysis was conducted with the column at 30 °C, an autosampler temperature of 4 °C, and a 5 µL injection volume.

An mass spectrometer (AB SCIEX 6500 QTRAP+, Framingham, MA, USA) triple quadrupole mass spectrometer with an electrospray ionization (ESI) interface was used for the assay in the multiple reaction monitoring (MRM) mode. The ion source parameters were set as follows: ionspray voltage, −4500 V; temperature, 450 °C; ion source gas 1, 45 psi; ion source gas 2, 45 psi; curtain gas, 30 psi. The MRM parameters for each of the targeted analytes were optimized using flow injection analysis, by injecting the standard solutions of the individual analytes, into the API source of the mass spectrometer.

### 2.7. Statistical Analysis

Statistical analysis was performed using IBM SPSS Statistics 26 (IBM, Armonk, NY, USA). One-way ANOVA with Duncan’s multiple range test was applied to compare FAV, ROS contents, and antioxidant enzyme activities in rice grains, with significance defined at *p* < 0.05. Origin 2022 software (Origin-Lab, Northampton, MA, USA) was used to draw figures.

The raw metabolomics data were imported into MetaboAnalyst 5.0 (https://www.metaboanalyst.ca/, accessed on 5 September 2025) for preprocessing, including filtering of deviations, missing value imputation, and normalization. Subsequently, multivariate statistical analysis, including principal component analysis (PCA), heatmap cluster analysis (HCA) and orthogonal partial least squares discriminant analysis (OPLS-DA) were conducted using MetaboAnalyst 5.0 and SIMCA 14.1. The cluster heatmaps were drawn using R. Differential metabolites were functionally annotated against the Oryza sativa japonica database in the Kyoto Encyclopedia of Genes and Genomes (KEGG, https://www.genome.jp/kegg/, accessed on 5 September 2025), followed by comparative analysis of KEGG metabolic pathways. The metabolic pathway map was drawn using Adobe Illustrator 2020.

## 3. Results

### 3.1. Effect of N_2_-MAS on Fatty Acid Values (FAV) and Reactive Oxygen Species (ROS) in Rice Grains

**Figure 1:**
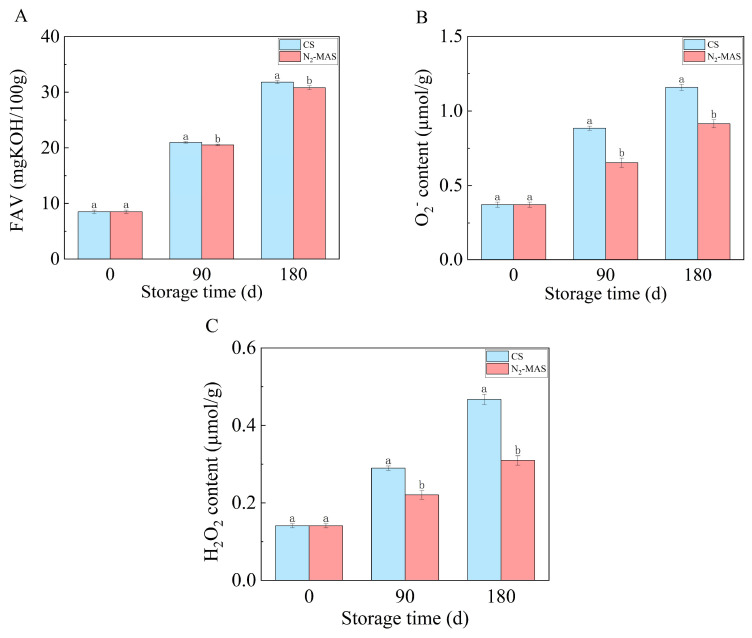
Changes in FAV and ROS contents of CS and N_2_-MAS rice grains. (**A**) FAV, (**B**) O_2_^−^ content, (**C**) H_2_O_2_ content. (a, b) indicated significant differences at the *p* < 0.05 level. CS denotes conventional storage. N_2_-MAS denotes nitrogen-modified atmosphere storage.

As shown in Figure 1A, the fatty acid values (FAV) of rice grains revealed an increasing trend throughout storage in both N_2_-MAS and CS. Comparative analysis revealed that N_2_-MAS rice grains exhibited significantly lower FAV concentrations during storage compared to CS (*p* < 0.05). At the end of the storage, the FAV of rice grains in CS was 31.83 mgKOH/100 g and that in N_2_-MAS was 30.80 mgKOH/100 g.

Figure 1B,C illustrate the changes in ROS levels between N_2_-MAS and CS rice grains during the storage, which both increased after 180 days of storage. It was notable that the contents of O_2_^−^ and H_2_O_2_ in the N_2_-MAS rice grains were lower than that in the CS at the same storage time. At the end of storage, the O_2_^−^ and H_2_O_2_ contents in CS-stored rice grains were quantified at 1.16 µmol·g^−1^ and 0.47 µmol·g^−1^, respectively. While those in N_2_-MAS rice grains measured 0.91 µmol·g^−1^ and 0.31 µmol·g^−1^. Therefore, N_2_-MAS rice grains exhibited comparatively lower level (*p* < 0.05) of ROS.

### 3.2. Effect of N_2_-MAS on the Activities of Antioxidant Enzymes

**Figure 2:**
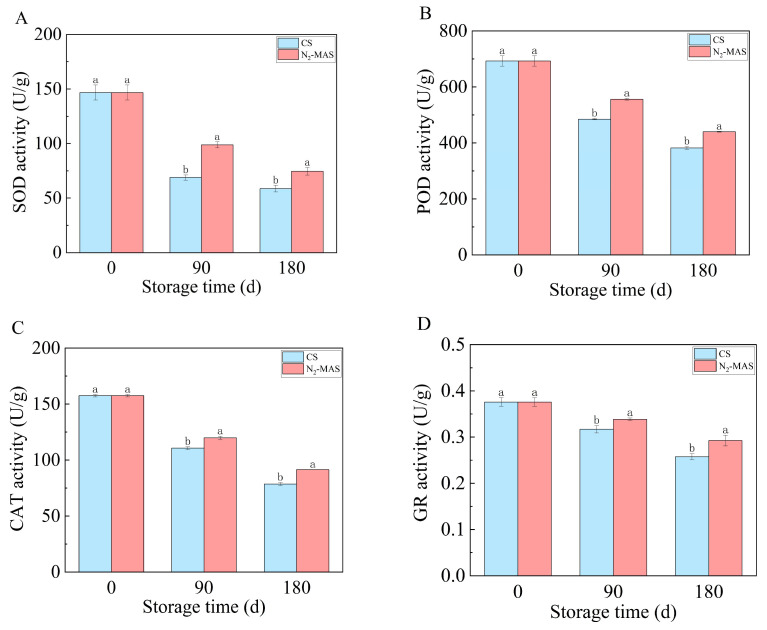
Changes in activities of antioxidant enzymes of CS and N_2_-MAS rice grains. (**A**) SOD activity, (**B**) POD activity, (**C**) CAT activity, (**D**) GR activity. (a, b) indicated significant differences at the *p* < 0.05 level. CS denotes conventional storage. N_2_-MAS denotes nitrogen-modified atmosphere storage.

As revealed in Figure 2A,D, the activities of SOD, POD, CAT and GR in N_2_-MAS and CS rice grains significantly decreased during storage. All these four enzymes in the N_2_-MAS rice grains had significantly higher activities compared to the CS during storage (*p* < 0.05).

### 3.3. Effect of N_2_-MAS on the Contents of Pyridine Nucleotides in Rice Grains

**Figure 3:**
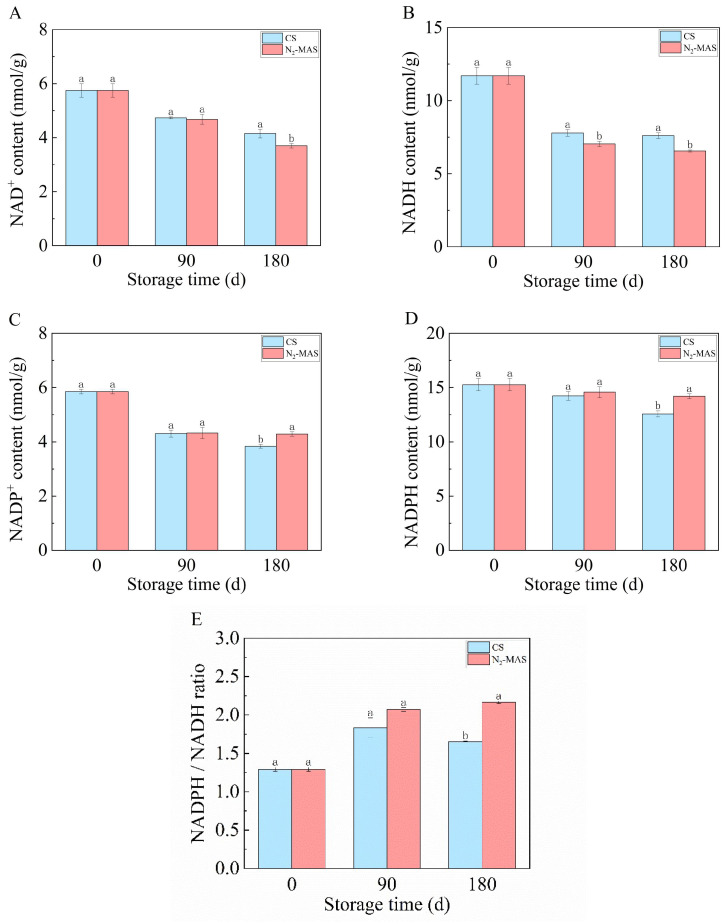
Changes in contents of pyridine nucleotides of CS and N_2_-MAS rice grains. (**A**) NAD^+^ content, (**B**) NADH content, (**C**) NADP^+^ content, (**D**) NADPH content. (**E**) NADPH/NADH ratio. (a, b) indicated significant differences at the *p* < 0.05 level. CS denotes conventional storage. N_2_-MAS denotes nitrogen-modified atmosphere storage.

During the storage, both CS and N_2_-MAS rice grains exhibited a progressive decline in NAD^+^ content, with N_2_-MAS rice grains consistently demonstrating significantly lower NAD^+^ levels compared to CS (Figure 3A). A parallel trend was observed in NADH dynamics, where both storage conditions showed a rapid depletion of NADH during the initial three months of storage, followed by a gradual reduction from 90 to 180 days. Notably, N_2_-MAS rice grains maintained substantially lower NADH concentrations throughout the storage duration relative to its CS counterpart (Figure 3B).

During storage, both CS and N_2_-MAS rice grains displayed a sharp decline in NADP^+^ content within the first three months, followed by a slower decrease from 90 to 180 days, with N_2_-MAS ultimately exhibiting higher NADP^+^ levels than CS at 180 days (Figure 3C). Concurrently, NADPH content decreased in both CS and N_2_-MAS rice grains, with N_2_-MAS maintaining significantly higher levels than CS after 180 days despite their shared declining trends (Figure 3D).

As revealed in Figure 3E, the NADPH/NADH ratio exhibited a progressive increase during the storage. Furthermore, N_2_-MAS grains displayed a significantly elevated NADPH/NADH ratio compared to CS grains at the end of storage.

### 3.4. Effect of N_2_-MAS on the Metabolites in Rice Grains

#### 3.4.1. Comparative Metabolomic Profiling of Central Carbon Metabolites in Rice Grains Under N_2_-MAS and CS

In our study, targeted metabolomics was employed to analyze the 55 metabolites associated with central carbon metabolism, with each group of samples comprising four biological replicates (Table S1). A total of 27 metabolites in rice grains were identified. Further statistical analysis of the results is shown in Table 1.

#### 3.4.2. Multivariate Statistical Analysis

To gain more insight into the relationship between metabolites and the central carbon metabolites metabolic mode of the stored rice grains, principal component analysis (PCA), heatmap cluster analysis (HCA), and orthogonal projections to latent structures–discriminant analysis (OPLS–DA) were carried out.

**Figure 4:**
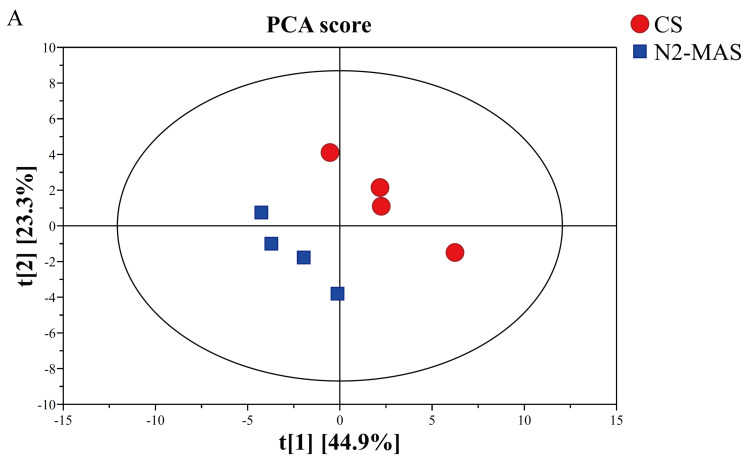

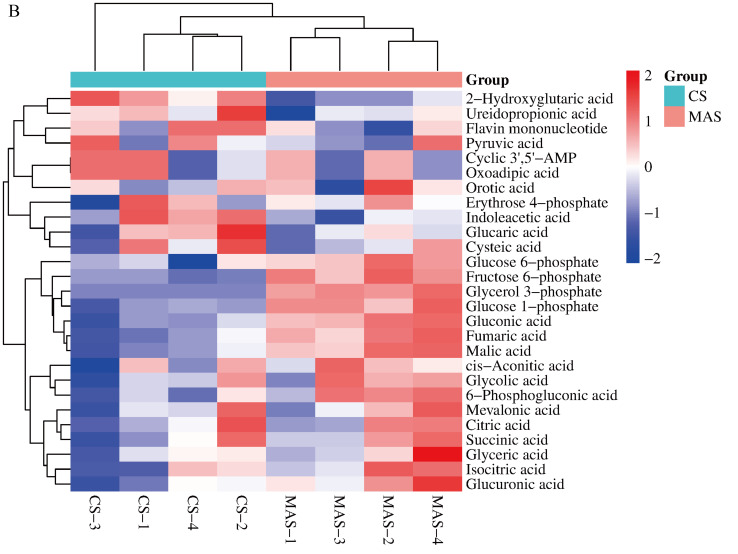

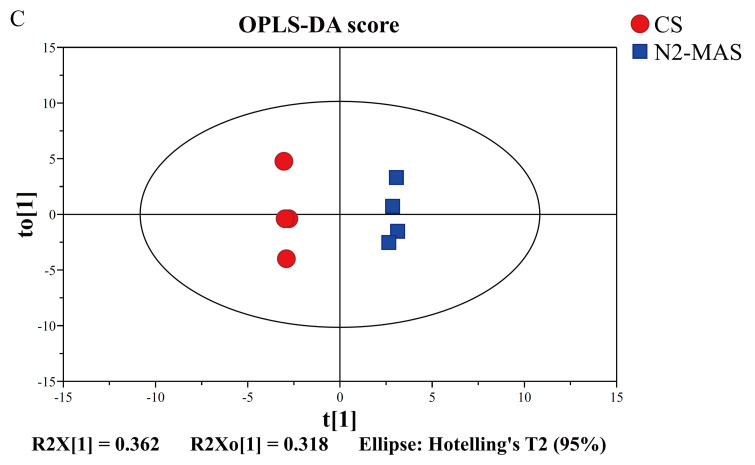

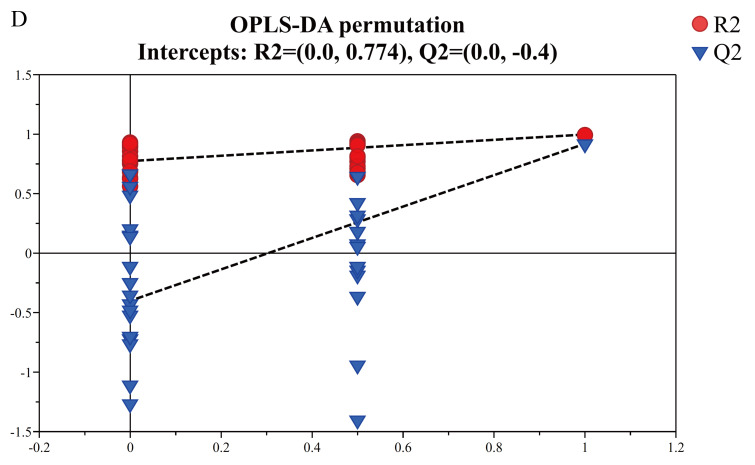

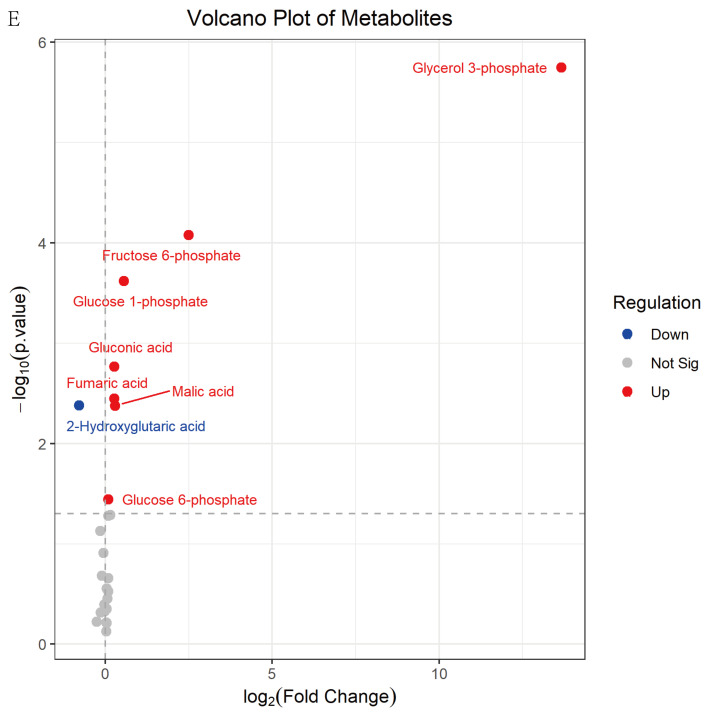

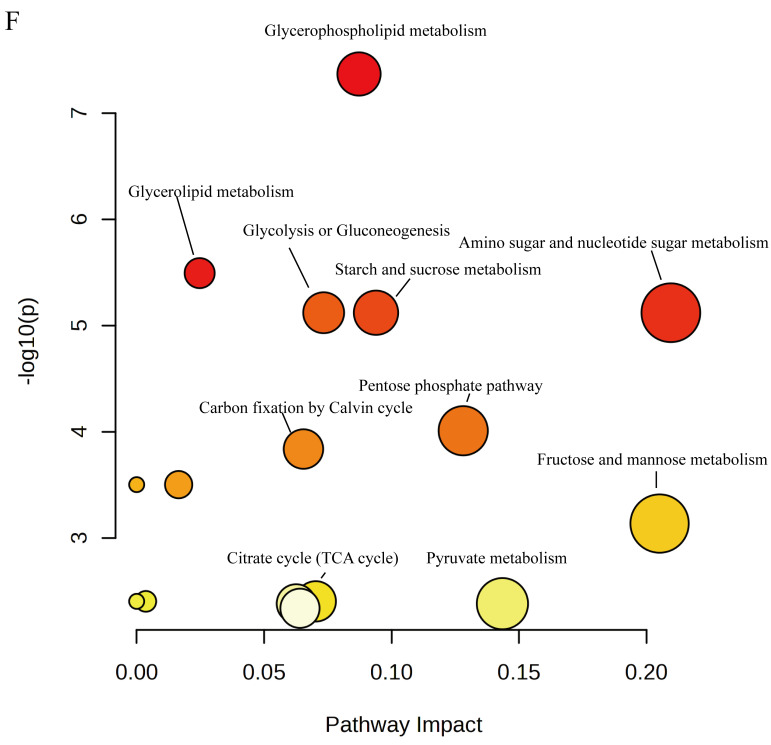
(**A**) PCA score plot. Each scatter referred to one determination. Different scatter shapes and colors indicate different groups. (**B**) HCA of metabolites between CS and N_2_-MAS rice grains, the color red and blue represent up-regulated and down-regulated. (**C**) OPLS–DA score plot. (**D**) Permutation test of OPLS–DA. (**E**) Volcano plot of metabolites. (**F**) KEGG pathway analysis. CS denotes conventional storage. N_2_-MAS denotes nitrogen-modified atmosphere storage.

To identify the differential metabolites among different storage conditions, the PCA and HCA analyses were first performed. As an unsupervised multivariate statistical analysis method, PCA could be utilized to examine metabolic differences in rice grains between N_2_-MAS and CS. The principal component score plot (Figure 4A) was based on a 95% confidence interval level. PC1 and PC2 represented the first and second principal components, respectively. PCA score plot demonstrated clear metabolic differentiation between CS and N_2_-MAS rice grains groups. The first two principal components (PC1 and PC2) collectively explained 68.2% of the total variance, with individual contributions of 44.9% and 23.3%, respectively. Consistent with the PCA result, the HCA (Figure 4B) also revealed that the metabolites between the CS and N_2_-MAS rice were obviously different. Collectively, the PCA and HCA results revealed markedly distinct metabolite profiles between the N_2_-MAS and CS rice groups.

To further optimize the separation between CS and N_2_-MAS rice grains, the supervised statistical method OPLS–DA was used to further analyze the two sets of samples (Figure 4C). The employed OPLS–DA model was rigorously validated, with key parameters of R^2^Y = 0.996 and Q^2^ = 0.956. As shown in Figure 4C, CS and N_2_-MAS rice grains showed a clear separation using OPLS–DA method. It revealed significantly changed metabolic pattern in N_2_-MAS rice grains compared to CS. During the modeling process, a permutation test was employed to prevent overfitting in the supervised model and ensure its validity. The result of the 200 permutation tests is shown in Figure 4D, with intercepts of R^2^ = 0.774 and Q^2^ = −0.4, the original Q^2^ value was significantly higher than the distribution of permuted Q^2^ values, thereby confirming the validity and reliability of the OPLS-DA model. These results confirmed that the metabolite differences between the groups revealed by OPLS-DA were statistically significant. The variable importance in the projection (VIP) obtained from OPLS-DA were annotated in Table 1. VIP scores exceeding 1.0 were identified as biologically significant differential metabolites [14].

#### 3.4.3. Identification of Key Metabolites in Rice Grains

To identify metabolites with substantial variations and statistically significant differences, the criteria of “VIP > 1, FC > 1.2 or FC < 0.8333, and *p* < 0.05” were applied to screen for metabolites that exhibited significant changes in N_2_-MAS to CS. 7 significant differential metabolites were identified (Table 1), including fructose 6-phosphate, glycerol 3-phosphate, glucose 1-phosphate, gluconic acid, 2-hydroxyglutaric acid, fumaric acid, and malic acid. Among these metabolites, six metabolites (fructose 6-phosphate, glucose 1-phosphate, glycerol 3-phosphate, gluconic acid, fumaric acid, malic acid) were up-regulated and one metabolite (2-hydroxyglutaric acid) was down-regulated in N_2_-MAS rice grains compared to CS. To better visualize the identification results of differential metabolites, a volcano plot was generated based on all 27 metabolites (Figure 4E).

Pathway analysis based on the KEGG database was performed on the differential metabolites to explore their correlations, with the results presented in bubble maps to show the metabolic variations between N_2_-MAS and CS (Figure 4F). Each bubble corresponded to a specific metabolic pathway. The results indicated that N_2_-MAS influenced rice quality primarily by remodeling central carbon and energy metabolism, including glycolysis, the pentose phosphate pathway, and the TCA cycle, along with perturbations in amino sugar and nucleotide sugar, starch and sucrose, and glycerophospholipid metabolism.

## 4. Discussion

### 4.1. N_2_-MAS Induced Alterations in Oxidative Stability of Rice Grains

Reactive oxygen species (ROS), including superoxide (O_2_^−^) and hydrogen peroxide (H_2_O_2_), were routine products of cellular metabolic processes [15]. It was reported that ROS played a key role in regulating senescence in seeds; low concentration of ROS can be used as signaling transduction molecules coping with stress, promoting dormancy release and inducing seed germination, while excessive ROS accumulation could cause seed deterioration, lower germination rates and loss of viability during storage [8]. ROS is mainly produced by non-enzymatic processes, such as lipid peroxidation, but oxidoreductase enzymes such as mitochondrial electron transport chain (ETC) could also participate [16].

During seed aging, the gradual accumulation of ROS attacks on polyunsaturated fatty acids in cell membrane phospholipids lead to the continuous breakdown of long-chain fatty acids into smaller compounds. This process induces cellular lipid peroxidation, generating a significant amount of free fatty acids (FFAs), which may serve as one of the contributing factors to the elevated fatty acid values (FAV) in stored rice grains [15]. To scavenge ROS, SOD universally catalyzes the conversion of superoxide radicals into hydrogen peroxide (H_2_O_2_) in all aerobic organisms. Subsequently, H_2_O_2_ is further decomposed into molecular oxygen and H_2_O via the enzymatic activities of POD and CAT [17]. Furthermore, GR is essential for sustaining the availability of reduced glutathione (GSH). GSH plays an important role in scavenging ROS [18]. In summary, these enzymes (POD, CAT, SOD, GR) help cells eliminate free radicals, mitigate lipid peroxidation, and preserve cell membrane stability. Therefore, maintaining the activities of these antioxidant enzymes is a prerequisite for maintaining low levels of ROS, preserving seed vitality and ensuring long-term storage [19].

In our study, the contents of ROS (O_2_^−^ and H_2_O_2_) of the stored rice grains exhibited an escalating trend, and the activities of SOD, POD, CAT and GR decreased in both N_2_-MAS and CS rice grains. Compared with CS, rice grains in N_2_-MAS showed lower levels of ROS, and high activities of SOD, POD, CAT, and GR. These data demonstrated that N_2_-MAS maintained the activities of antioxidant system enzymes in plants to scavenge ROS. Therefore, N_2_-MAS effectively delayed ROS accumulation during rice grains storage, thereby alleviating ROS-induced damaging effects and inhibiting lipid peroxidation.

In plant cells, pyridine nucleotides (NAD^+^, NADP^+^, NADH, and NADPH) serve as essential cofactors, extensively involved in regulating cellular metabolism and electron transport processes [20]. NADPH serves as a key cofactor in the cellular antioxidant defense system, scavenging H_2_O_2_ through the regeneration of glutathione and the antioxidant protein thioredoxin. NADH, another crucial cofactor in cells, primarily participates in the electron transport chain (ETC), generating ATP through oxidative phosphorylation [7]. Furthermore, NAD^+^ can be catalyzed by NADK to generate NADP^+^, established a dynamic regulatory bridge between NADH and NADPH [7]. In the present work, after 180 days of storage, the N_2_-MAS rice grains exhibited significantly lower NADH levels, but higher NADPH levels compared to the CS. There was a significantly higher level in NADPH/NADH ratio in N_2_-MAS rice grains than that in CS (Figure 3E). These data indicated that N_2_-MAS enhanced NADPH generation and provided the reducing power required for rice cell antioxidation. Compared with CS, the delayed oxidative damage in rice grains under N_2_-MAS was likely attributed to the shift from NADH-related metabolic pathways to NADPH-related ones in hypoxic environments [21]. In CS rice grains, insufficient NADPH supply led to the inefficiency of the antioxidant system, accumulated oxidative damage, and accelerated quality deterioration [7].

### 4.2. N_2_-MAS-Induced Alterations in Central Carbon Metabolism and Its Relationships to Antioxidant System of Rice Grains

**Figure 5:**
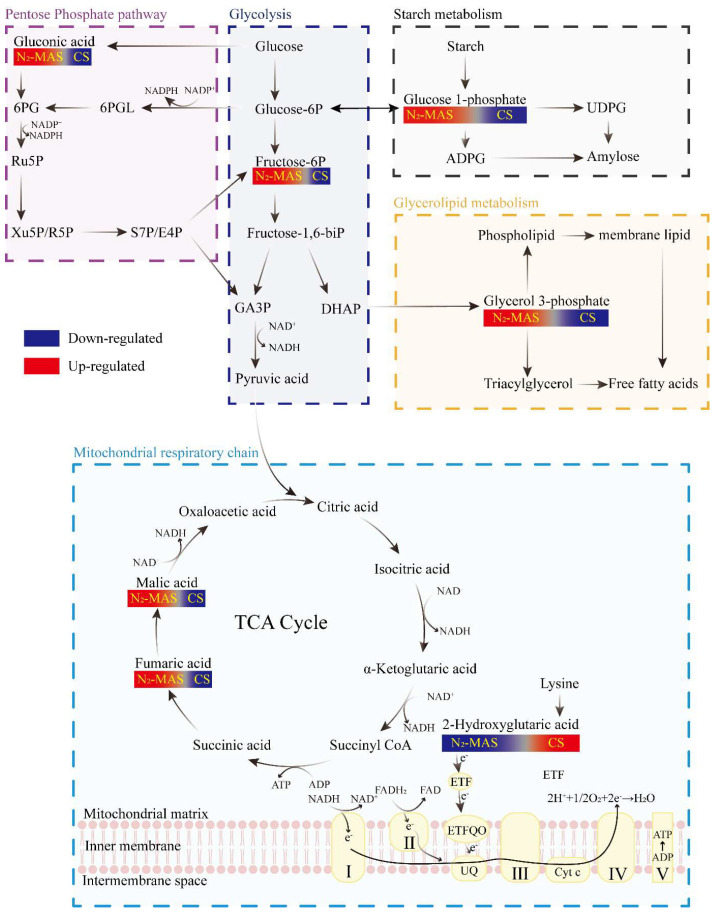
Mapping of differential metabolites onto central carbon metabolic pathways in rice grains under N_2_-MAS and CS. Metabolites increased or decreased in N_2_-MAS relative to CS are highlighted in red and blue, respectively. N_2_-MAS, nitrogen-modified atmosphere storage; CS, conventional storage. Abbreviations: Glucose-6P, glucose 6-phosphate; fructose-6P, fructose 6-phosphate; fructose-1,6-biP, fructose 1,6-bisphosphate; GA3P, glyceraldehyde-3phosphate; DHAP, dihydroxyacetone phosphate; 6PGL, 6-phosphogluconolactone; 6PG, 6-phosphogluconic acid; Ru5P, ribulose-5-phosphate; Xu5P, xylulose-5-phosphate; R5P, ribose-5-phosphate; S7P, sedoheptulose-7-phosphate; E4P, erythrose-4-phosphate; ADPG, adenosine diphosphate glucose; UDPG, uridine diphosphate glucose; ETF, electron transfer flavoprotein; ETFQO, electron transfer flavoprotein-Quinone Oxidoreductase; UQ, ubiquinone; complex I–V, mitochondrial electron transport chain complex.

The metabolomics profiling showed the significant differences between N_2_-MAS and CS rice grains in metabolites. To highlight the associations of the differential metabolites with central carbon metabolism, a network diagram was created (Figure 5).

Our study revealed that compared to CS, N_2_-MAS rice grains exhibited significant up-regulation of fructose-6-phosphate (F6P) and gluconic acid. F6P served as the key intermediate metabolite of glycolysis and one of the end products of PPP. In the glycolytic process, F6P was generated from glucose-6-phosphate through the catalytic action of phosphoglucose isomerase. Subsequently, this metabolite underwent further phosphorylation by phosphofructokinase, with the consumption of one ATP molecule, to yield fructose-1,6-bisphosphate, where it entered downstream glycolytic steps that contributed to NADH production [22]. Gluconic acid was primarily produced through the oxidation of glucose, and then was catalyzed by gluconokinase to generate 6-phosphogluconic acid (6-PG) [23]. Therefore, the decrease in NADH content, along with the accumulation of F6P and G1P collectively demonstrated that glycolysis in N_2_-MAS rice grains was inhibited at an intermediate stage. Glycolysis functioned as a critical upstream process for mitochondrial respiratory chain activity and served as one of the primary sources of NADH and ATP [24,25]. The mitochondrial respiratory chain was one of the major sources of cellular ROS [16]. Meanwhile, PPP was the key route for NADPH generation, which provided essential reducing power for antioxidant systems [7]. Therefore, the metabolic results showed that N_2_-MAS prevented excessive accumulation of ROS in rice grains cells (Figure 1B,C) by inhibiting glycolysis to restrict mitochondrial respiratory chain activity, while simultaneously elevating PPP-mediated NADPH biosynthesis (consistent with the results in Figure 3D). Such metabolic coordinated regulation in N_2_-MAS rice grains effectively mitigated oxidative damage caused by excessive ROS in grain cells.

The contents of fumaric acid and malic acid, as TCA cycle intermediates, were significantly increased in N_2_-MAS rice grains compared to CS. Previous studies had demonstrated that such TCA cycle intermediates could improve plant abiotic stress tolerance [26]. It was reported that the up-regulation of malic acid and fumaric acid played important roles in nano-selenium-induced improvement in antioxidant capacity in melon. The elevated levels of malic acid and fumaric acid could support increased activity of antioxidant enzymes, forming a synergistic antioxidant network [27]. Our observation suggested that N_2_-MAS improved abiotic stress tolerance of rice grains and internal ROS homeostasis regulation by enhancing fumaric acid and malic acid accumulation.

The concentration of glycerol 3-phosphate in the CS rice was below the limit of quantification for this metabolite, indicating that its level in the CS rice was extremely low and fell below the threshold for accurate instrumental quantification. Nevertheless, the signal detected for glycerol 3-phosphate in the N_2_-MAS group was clear and reliable. This result strongly suggested that glycerol 3-phosphate metabolism was significantly up-regulated in N_2_-MAS rice. G3P served as an essential substrate for triacylglycerol (TAG) biosynthesis and played a critical role in regulating glycerolipid biosynthesis [28]. Additionally, previous studies reported that G3P served as a critical precursor for intracellular phospholipid synthesis and was directly involved in membrane lipid biogenesis [29]. The accumulation of G3P contributed to modulating cellular ROS levels and enhancing antioxidant stress tolerance [30]. In our experiment, N_2_-MAS up-regulated G3P levels in rice grains, thereby enhancing membrane lipid stability and repair capacity, ultimately reduced free fatty acids and ROS release during storage resulting from membrane lipid degradation, which was in accordance with the results displayed in Figure 1.

Metabolomic analysis also revealed that the content of 2-hydroxyglutarate (2-HG), a five-carbon dicarboxylic acid involved in lysine degradation [31], was significantly down-regulated in N_2_-MAS rice grains compared to CS. Previous studies reported that lysine could serve as a substrate for 2-HG biosynthesis in plants [32]. It had been demonstrated that 2-HG was catalyzed to 2-oxoglutarate (2-OG) by the ETF/ETFQO enzyme complex, which acted as an electron acceptor in this reaction [31]. The ETT/ETFQO complex comprised the electron transfer flavoprotein (ETF) and the electron-transfer flavoprotein: ubiquinone oxidoreductase (ETFQO), which served as a key component of the mitochondrial electron transport chain (ETC), was responsible for transferring electrons generated from fatty acid β-oxidation to the UQ in ETC [32]. 2-HG was shown to accumulate abnormally in plants with dysfunctional EFT/ETFQO complexes [33]. These observations indicated that 2-HG accumulation could serve as a biomarker for cellular metabolic dysfunction. Collectively, the reduced 2-HG levels in N_2_-MAS rice grains suggested that N_2_-MAS effectively prevented damage to the mitochondrial ETC enzyme complexes, thereby maintaining mitochondrial functional integrity.

Our data showed that glucose-1-phosphate (G1P) level in N_2_-MAS rice grains was significantly up-regulated compared to CS. G1P was primarily synthesized through starch phosphorolysis catalyzed by starch phosphorylase [34]. Additionally, G1P and the glycolytic intermediate glucose-6-phosphate (Glucose-6P) were enzymatically interconvertible catalyzed by phosphoglucomutase [35]. It had been shown that G1P serves as a substrate for the enzymatic formation of adenosine diphosphate glucose (ADPG) and uridine diphosphate glucose (UDPG), both of which are key precursors for amylose biosynthesis [36]. It was reported that nitrogen-modified atmosphere storage could retard the increase in amylose content [2]. Located at the metabolic intersection of glycolysis and starch metabolism, G1P plays pivotal roles in energy storage, cellular structure maintenance, and stress adaptation (Figure 5). Previous studies demonstrated that plants up-regulated G1P levels to enhance stress tolerance under abiotic stress. For instance, drought-stressed quinoa seeds and waterlogged cotton plants showed significant G1P up-regulation [37,38]. Consistent with our experiment, it suggested that N_2_-MAS-induced G1P accumulation was associated with the enhancement of hypoxia stress tolerance and the inhibition of amylose content increase in rice grains.

In summary, N_2_-MAS suppressed respiratory metabolic activity in rice grains, thereby preventing ROS excessive accumulation caused by respiratory metabolism. Concurrently, it enhanced the PPP flux, thereby promoting NADPH biosynthesis and synergizing with the antioxidant system to maintain ROS homeostasis in rice grains cells. Furthermore, N_2_-MAS elevated stress tolerance-associated metabolites, including malic acid, fumaric acid, G3P, and G1P, sustaining cellular homeostasis and delaying quality deterioration during storage.

## 5. Conclusions

This study demonstrated that N_2_-MAS effectively mitigated the increase in fatty acid values (FAV) in rice grains by modulating key metabolic pathways. As FAV serves as a key indicator for evaluating the quality stability of rice grain and its processed products, the increase in FAV was typically accompanied by the development of off-flavors and a decline in eating quality. Significant alterations were observed in metabolites linked to antioxidant defense systems, respiratory metabolism, and glycerollipid metabolism, indicating that N_2_-MAS effectively regulated the metabolic network related to lipid degradation at the molecular level. Furthermore, N_2_-MAS maintained NADPH generation and significantly reduced the accumulation of reactive oxygen species such as O_2_^−^ and H_2_O_2_ by suppressing respiratory metabolism and promoting the pentose phosphate pathway in rice grains, thereby better preserving the original flavor and textural properties of rice during prolonged storage. Additionally, N_2_-MAS up-regulated G3P, G1P, malic acid, and fumaric acid synthesis to enhance the tolerance of rice grains to hypoxic storage. These findings not only provide new insights into the hypoxic response mechanisms of grain storage but also establish a theoretical foundation for the application of nitrogen-modified atmosphere storage in the commercial preservation of rice.

## Figures and Tables

**Table 1:** Differential metabolites were identified between CS and N_2_-MAS rice grains after 180 days storage.

Metabolites	Mean (nmol/g)		*p*-Value	VIP	Fold Change
	N_2_-MAS	CS			N_2_-MAS/CS
Malic acid	19.2	15.8	0.00	1.33	1.22
Glycerol 3-phosphate	65.3	-	0.00	1.45	+∞
Glucose 1-phosphate	0.185	0.126	0.00	1.38	1.47
Gluconic acid	180	150	0.00	1.36	1.20
Fumaric acid	7.86	6.55	0.00	1.33	1.20
Fructose 6-phosphate	28.0 × 10^−3^	5.00 × 10^−3^	0.00	1.36	5.61
2-Hydroxyglutaric acid	3.98	6.91	0.00	1.29	0.58

VIP denotes the variable importance in the projection from OPLS-DA. *p*-value was derived from *t*-test.

## Data Availability

The original contributions presented in this study are included in the article/Supplementary Materials. Further inquiries can be directed to the corresponding author.

## Supplementary Materials

The following supporting information can be downloaded at https://www.mdpi.com/article/10.3390/foods14213643/s1, Table S1: Quantitative results of 55 targeted metabolites; Table S2: Targeted Analyte Information; Figure S1: Chromatograms of standards (A) and samples (B).
