# Supplementary material for: Study on the Changes of Antioxidant System and Respiratory Metabolism in Rice Grains Under Nitrogen-Modified Atmosphere Storage from the Targeted Metabolomics Perspective
Source: Foods. 2025 Oct 25;14(21):3643. doi: 10.3390/foods14213643 (PMC12610211; doi:10.3390/foods14213643)
Supplement: Supplementary file 1 [file foods-14-03643-s001.zip › foods-3908273-supplementary.pdf]

Table S1. Quantitative results of 55 targeted metabolites (nmol/g).

Compound Name	Conventional storage	Nitrogen-modified atmosphere storage
2-Aminobenzoic acid	NA	NA
2-Hydroxyglutaric acid	6.91±0.83	3.98±0.77
2-Isopropylmalic acid	NA	NA
2-Ketobutyric acid	NA	NA
3-Phosphoglyceric acid	NA	NA
4-Hydroxyphenylpyruvic acid	NA	NA
6-Phosphogluconic acid	1.83±0.05	1.93±0.05
Adenosine diphosphate	NA	NA
Adenosine triphosphate	NA	NA
alpha-Ketoglutaric acid	NA	NA
alpha-Ketoisovaleric acid	NA	NA
cis-Aconitic acid	1.50±0.06	1.55±0.03
Citric acid	125±6	127±6
Cyclic 3',5'-AMP	0.00506±0.00202	0.00421±0.00165
Cysteic acid	3.36±0.09	3.31±0.06
Dihydroxyacetone phosphate	NA	NA
Erythrose 4-phosphate	4.01±0.19	4.07±0.06
Flavin mononucleotide	0.0721±0.0045	0.0669±0.0040
Flavine-adenine dinucleotide	NA	NA
Fructose 1,6-bisphosphate	NA	NA
Fructose 6-phosphate	0.00506±0.00202	0.0283±0.0022
Fumaric acid	6.55±0.40	7.86±0.29
Galacturonic acid	NA	NA
Glucaric acid	1.03±0.05	1.00±0.02
Gluconic acid	150±8	180±6
Glucose 1-phosphate	0.126±0.009	0.185±0.010
Glucose 6-phosphate	0.0758±0.0029	0.0806±0.0011
Glucuronic acid	1.90±0.09	2.09±0.10
Glyceric acid	32.3±1.1	33.7±2.0
Glycerol 3-phosphate	NA	65.3±6.2
Glycolic acid	13.4±0.84	14.1±0.8
Glyoxylic acid	NA	NA
Guanosine diphosphate	NA	NA
Guanosine triphosphate	NA	NA
Hippuric acid	NA	NA
Homogentisic acid	NA	NA
Indoleacetic acid	2.78±0.19	2.50±0.13
Isocitric acid	6.03±0.35	6.42±0.33
Maleic acid	NA	NA
Malic acid	15.8±1.0	19.2±0.8
Mannose 6-phosphate	NA	NA
Methylmalonic acid	NA	NA

Mevalonic acid	0.507±0.036	0.522±0.031
Orotic acid	0.0924±0.0043	0.0943±0.0085
Oxoadipic acid	0.00506±0.00202	0.00421±0.00165
Phosphoenolpyruvic acid	NA	NA
Picolinic acid	NA	NA
Pyruvic acid	7.61±1.19	6.90±1.14
Quinolinic acid	NA	NA
Ribose 5-phosphate	NA	NA
Ribulose 1,5-bisphosphate	NA	NA
Sedoheptulose 7-phosphate	NA	NA
Succinic acid	15.3±0.7	15.7±0.5
Ureidopropionic acid	0.509±0.013	0.488±0.016
Vanillylmandelic acid	NA	NA

"NA" indicated that the result was not detected.

Table S2. Targeted Analyte Information.

Compound Name	CAS	Molecular formula	Molecular weight	HMDB ID
Adenosine triphosphate	56-65-5	$C_{10}H_{16}N_5O_{13}P_3$	507.18	HMDB0000538
Adenosine diphosphate	58-64-0	$C_{10}H_{15}N_5O_{10}P_2$	427.20	HMDB0001341
Flavine-adenine dinucleotide	146-14-5	$C_{27}H_{33}N_9O_{15}P_2$	785.55	HMDB0001248
Pyruvic acid	127-17-3	$C_3H_4O_3$	88.06	HMDB0000243
alpha-Ketoglutaric acid	328-50-7	$C_5H_6O_5$	146.10	HMDB0000208
Guanosine diphosphate	146-91-8	$C_{10}H_{15}N_5O_{11}P_2$	443.20	HMDB0001201
Succinic acid	110-15-6	$C_4H_6O_4$	118.09	HMDB0000254
Guanosine triphosphate	86-01-1	$C_{10}H_{16}N_5O_{14}P_3$	523.18	HMDB0001273
Glyoxylic acid	298-12-4	$C_2H_2O_3$	74.04	HMDB0000119
Flavin mononucleotide	146-17-8	$C_{17}H_{21}N_4O_9P$	456.34	HMDB0001520
Phosphoenolpyruvic acid	138-08-9	$C_3H_5O_6P$	168.04	HMDB0000263
Glycerol 3-phosphate	57-03-4	$C_3H_9O_6P$	172.07	HMDB0000126
Glucose 1-phosphate	59-56-3	$C_6H_{13}O_9P$	260.13	HMDB0001586
2-Aminobenzoic acid	118-92-3	$C_7H_7NO_2$	137.14	HMDB0001123
2-Ketobutyric acid	600-18-0	$C_4H_6O_3$	102.09	HMDB0000005
Dihydroxyacetone phosphate	57-04-5	$C_3H_7O_6P$	170.06	HMDB0001473
Ribose 5-phosphate	3615-55-2	$C_5H_{11}O_8P$	230.11	HMDB0001548
Fumaric acid	110-17-8	$C_4H_4O_4$	116.07	HMDB0000134

alpha-Ketoisovaleric acid	759-05-7	$C_5H_8O_3$	116.12	HMDB0000019
Malic acid	97-67-6	$C_4H_6O_5$	134.09	HMDB0000156
Citric acid	77-92-9	$C_6H_8O_7$	192.12	HMDB0000094
Glycolic acid	79-14-1	$C_2H_4O_3$	76.05	HMDB0000115
Glucuronic acid	528-16-5	$C_6H_{10}O_7$	194.14	HMDB0000127
3-Phosphoglyceric acid	820-11-1	$C_3H_7O_7P$	186.06	HMDB0000807
Gluconic acid	526-95-4	$C_6H_{12}O_7$	196.16	HMDB0000625
Glyceric acid	473-81-4	$C_3H_6O_4$	106.08	HMDB0000139
Erythrose 4-phosphate	585-18-2	$C_4H_9O_7P$	200.08	HMDB0001321
Orotic acid	65-86-1	$C_5H_4N_2O_4$	156.10	HMDB0000226
Isocitric acid	320-77-4	$C_6H_8O_7$	192.12	HMDB0000193
Oxoadipic acid	3184-35-8	$C_6H_8O_5$	160.12	HMDB0000225
Galacturonic acid	25990-10-7	$C_6H_{10}O_7$	194.14	HMDB0002545
6-Phosphogluconic acid	921-62-0	$C_6H_{13}O_{10}P$	276.14	HMDB0001316
cis-Aconitic acid	585-84-2	$C_6H_6O_6$	174.11	HMDB0000072
Mevalonic acid	150-97-0	$C_6H_{12}O_4$	148.16	HMDB0000227
Cysteic acid	498-40-8	$C_3H_7NO_5S$	169.16	HMDB0002757
Homogentisic acid	451-13-8	$C_8H_8O_4$	168.15	HMDB0000130
Cyclic 3',5'-AMP	60-92-4	$C_{10}H_{12}N_5O_6P$	329.21	HMDB0000058
Mannose 6-phosphate	3672-15-9	$C_6H_{13}O_9P$	260.14	HMDB0001078
Glucose 6-phosphate	56-73-5	$C_6H_{13}O_9P$	260.14	HMDB0001401
Glucaric acid	87-73-0	$C_6H_{10}O_8$	210.14	HMDB0000663
Indoleacetic acid	87-51-4	$C_{10}H_9NO_2$	175.18	HMDB0000197
4-Hydroxyphenylpyruvic acid	156-39-8	$C_9H_8O_4$	180.16	HMDB0000707
Ribulose 1,5-bisphosphate	24218-00-6	$C_5H_{12}O_{11}P_2$	310.09	HMDB0244230
Maleic acid	110-16-7	$C_4H_4O_4$	116.07	HMDB0000176
Hippuric acid	495-69-2	$C_9H_9NO_3$	179.17	HMDB0000714
Methylmalonic acid	516-05-2	$C_4H_6O_4$	118.09	HMDB0000202
2-Isopropylmalic acid	3237-44-3	$C_7H_{12}O_5$	176.17	HMDB0000402

Ureidopropionic acid	462-88-4	$C_4H_8N_2O_3$	132.12	HMDB0000026
2-Hydroxyglutaric acid	13095-48-2	$C_5H_8O_5$	148.11	HMDB0000694
Quinolinic acid	89-00-9	$C_7H_5NO_4$	167.12	HMDB0000232
Fructose 6-phosphate	643-13-0	$C_6H_{13}O_9P$	260.14	HMDB0000124
Fructose 1,6-bisphosphate	34693-15-7	$C_6H_{14}O_{12}P_2$	340.11	HMDB0001058
Sedoheptulose 7-phosphate	2646-35-7	$C_7H_{15}O_{10}P$	290.16	HMDB0001068
Vanillylmandelic acid	55-10-7	$C_9H_{10}O_5$	198.17	HMDB0000291
Picolinic acid	98-98-6	$C_6H_5NO_2$	123.11	HMDB0002243

A

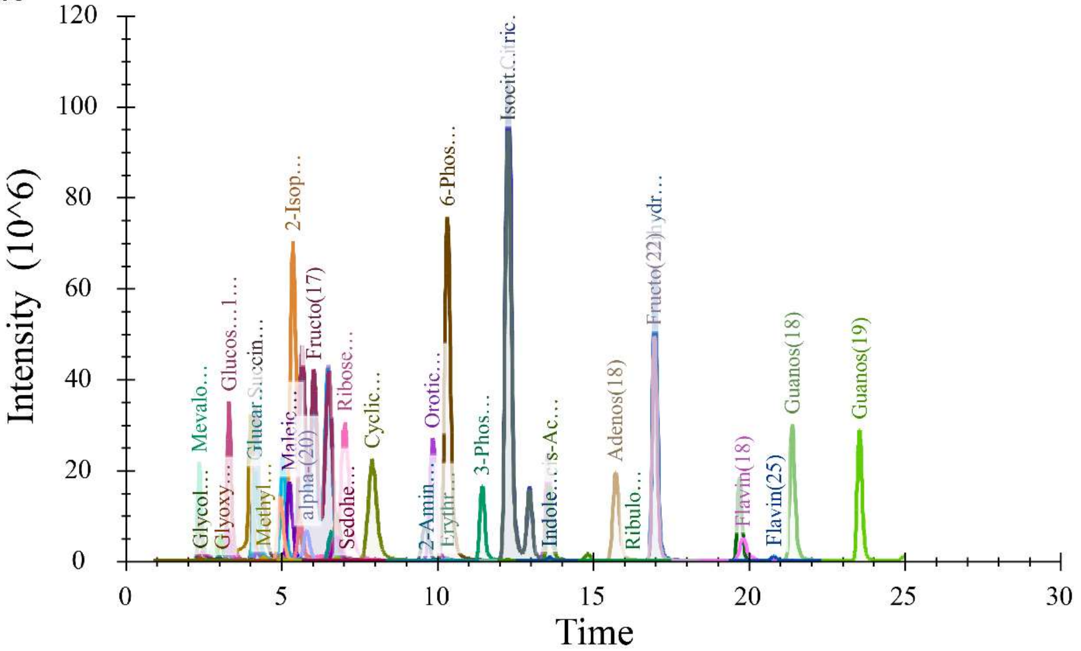

Intensity (10^6)
Time
0
20
40
60
80
100
120
0
5
10
15
20
25
30
Mevalo…
Glycol…
Glyoxy…
Glucos…1…
Glucar
Succin…
Methyl…
2-Isop…
Maleic…
alpha-(20)
Fructo(17)
Ribose…
Sedohe…
Cyclic…
Orotic…
2-Amin…
Erythr…
6-Phos…
3-Phos…
Indole…
cis-Ac…
Adenos(18)
Ribulo…
Fructo(22)
Flavin(18)
Flavin(25)
Guanos(18)
Guanos(19)

B

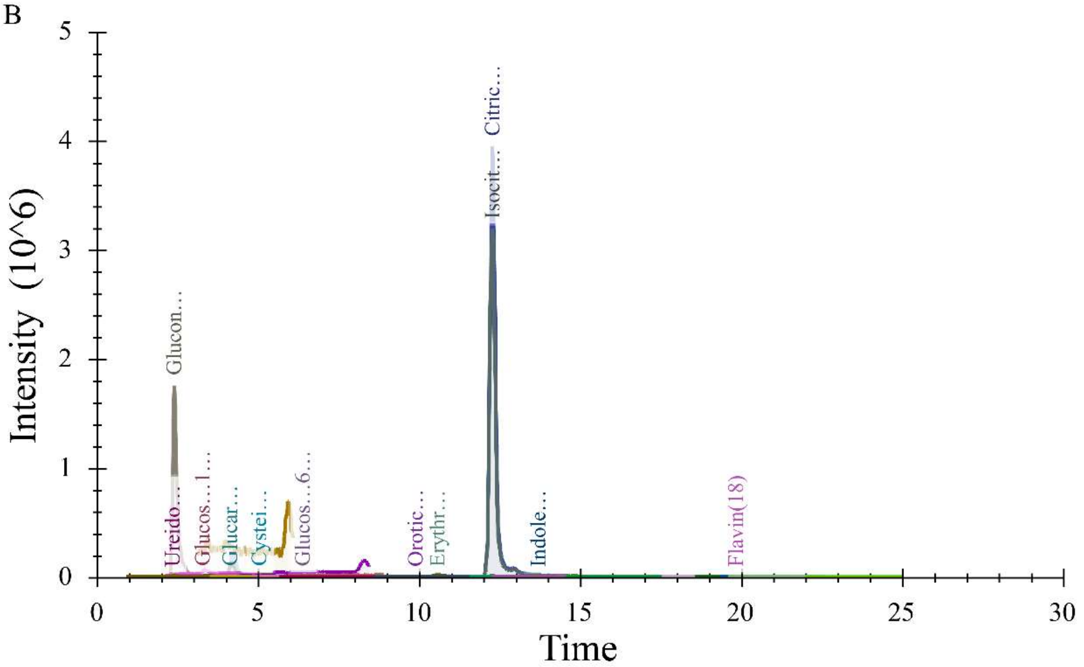

Intensity (10^6)
Time
0
1
2
3
4
5
0
5
10
15
20
25
30
Glucon…
Ureido…
Glucos…1…
Glucar…
Cystei…
Glucos…6…
Orotic…
Erythr…
Isocit…
Citric…
Indole…
Flavin(18)

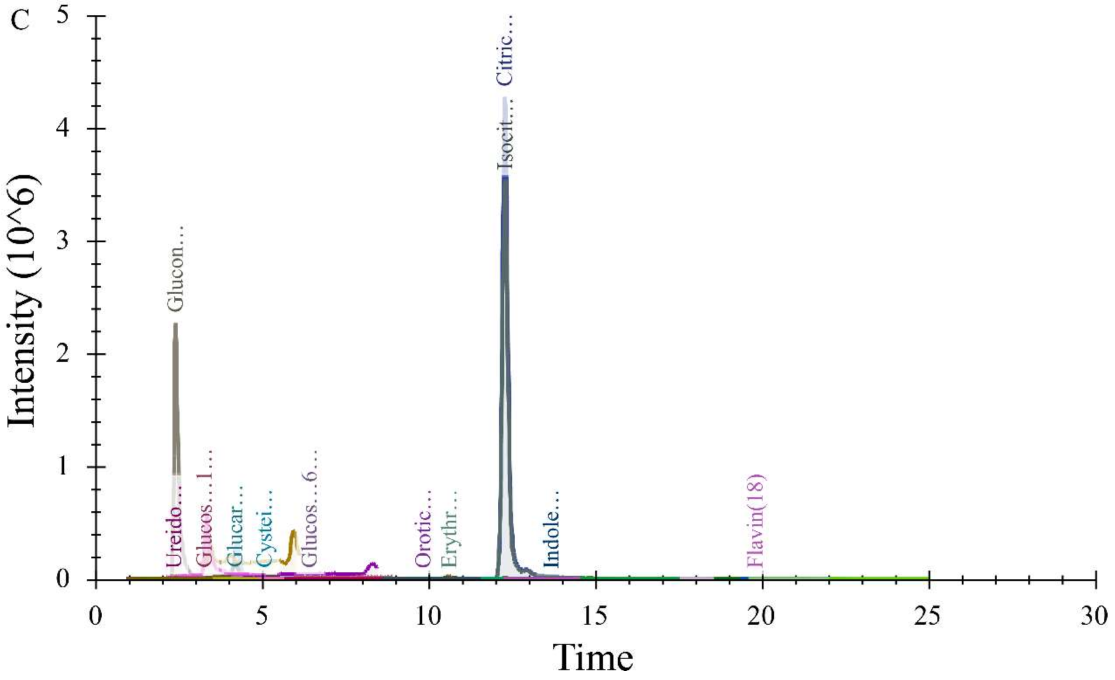

Figure S1. (A) Chromatograms of standards, (B) Chromatograms of CS rice grains, (C) Chromatograms of N_2-MAS rice grains.
